# Broad genomic and transcriptional analysis reveals a highly derived genome in dinoflagellate mitochondria

**DOI:** 10.1186/1741-7007-5-41

**Published:** 2007-09-27

**Authors:** Christopher J Jackson, John E Norman, Murray N Schnare, Michael W Gray, Patrick J Keeling, Ross F Waller

**Affiliations:** 1 School of Botany, the University of Melbourne, Victoria 3010, Australia; 2 Department of Biochemistry and Molecular Biology, Dalhousie University, Halifax, Nova Scotia, B3H 1X5, Canada; 3 Department of Botany, University of British Columbia, Vancouver, British Columbia, V6T 1Z4, Canada

## Abstract

**Background:**

Dinoflagellates comprise an ecologically significant and diverse eukaryotic phylum that is sister to the phylum containing apicomplexan endoparasites. The mitochondrial genome of apicomplexans is uniquely reduced in gene content and size, encoding only three proteins and two ribosomal RNAs (rRNAs) within a highly compacted 6 kb DNA. Dinoflagellate mitochondrial genomes have been comparatively poorly studied: limited available data suggest some similarities with apicomplexan mitochondrial genomes but an even more radical type of genomic organization. Here, we investigate structure, content and expression of dinoflagellate mitochondrial genomes.

**Results:**

From two dinoflagellates, *Crypthecodinium cohnii *and *Karlodinium micrum*, we generated over 42 kb of mitochondrial genomic data that indicate a reduced gene content paralleling that of mitochondrial genomes in apicomplexans, i.e., only three protein-encoding genes and at least eight conserved components of the highly fragmented large and small subunit rRNAs. Unlike in apicomplexans, dinoflagellate mitochondrial genes occur in multiple copies, often as gene fragments, and in numerous genomic contexts. Analysis of cDNAs suggests several novel aspects of dinoflagellate mitochondrial gene expression. Polycistronic transcripts were found, standard start codons are absent, and oligoadenylation occurs upstream of stop codons, resulting in the absence of termination codons. Transcripts of at least one gene, *cox3*, are apparently trans-spliced to generate full-length mRNAs. RNA substitutional editing, a process previously identified for mRNAs in dinoflagellate mitochondria, is also implicated in rRNA expression.

**Conclusion:**

The dinoflagellate mitochondrial genome shares the same gene complement and fragmentation of rRNA genes with its apicomplexan counterpart. However, it also exhibits several unique characteristics. Most notable are the expansion of gene copy numbers and their arrangements within the genome, RNA editing, loss of stop codons, and use of trans-splicing.

## Background

The origin of mitochondria by endosymbiosis has emerged as a pivotal event in the evolution of eukaryotes. All eukaryote groups that have been studied bear a derivative of this endosymbiont, and for most the resulting mitochondrion is central to energy metabolism as well as providing several other anabolic and catabolic functions [1]. A relict, though functionally essential, mitochondrial genome (or mtDNA) persists in all but a few anaerobic eukaryotes, and the genes in these genomes firmly identify the original endosymbiont as an α-proteobacterium [2]. The jakobid flagellate *Reclinomonas americana *has the least derived mitochondrial genome characterized to date, with at least 97 genes encoded on a single, circular-mapping 69 kb chromosome [3]. More typically mitochondrial genomes have been reduced to 40–50 genes arranged on either circular- or linear-mapping chromosomes of 15–60 kb (although many plant mitochondrial genomes have been secondarily expanded to several hundreds to thousands of kb) [4].

In some eukaryotic groups, however, the mtDNA has been modified more substantially, resulting in extremes in genome structure. For example, trypanosomatid mtDNA consists of a few dozen large circular molecules and several thousand minicircles that encode guide RNAs that participate in extensive U insertion/deletion RNA editing [5]. Diplonemid mitochondria also contain multiple circular mtDNA molecules, each encoding gene fragments that are trans-spliced to generate functional transcripts [6]. Another example is the mtDNA in the ichthyosporean, *Amoebidium parasiticum*: in this case, mitochondrial genes are fragmented and dispersed over several hundred linear chromosomes, totaling > 200 kb [7]. Over the diversity of eukaryotes, mitochondrial genomes exhibit other interesting characteristics, including the use of a number of different non-standard genetic codes, many of which involve alterations in start and, more rarely, stop codons [8,9].

One large group in which particularly interesting mitochondrial genome variation has been found is alveolates. Three major phyla make up alveolates: ciliates, apicomplexans, and dinoflagellates, with apicomplexans and dinoflagellates being sister clades to the exclusion of ciliates [10,11]. Within alveolates, ciliate mtDNA is the most conventional, consisting of a linear molecule, 40–50 kb in length, that codes for many of the standard mitochondrial proteins found in other organisms [12]. By contrast, the mtDNA of the apicomplexan genus *Plasmodium *is the smallest known, consisting of a linear, 6 kb tandem repeat [13] with only three protein-coding genes: cytochrome oxidase subunit 1 (*cox1*), cytochrome oxidase subunit 3 (*cox3*) and cytochrome b (*cob*). In addition, ciliate mtDNA encodes two ribosomal RNAs (rRNAs), but the corresponding apicomplexan genes are fragmented to an unprecedented degree and scattered about the genome [13,14].

To date, dinoflagellate mtDNAs have been the least well studied of alveolate mitochondrial genomes, with existing data pointing to a genome exhibiting several eccentricities. The first sequences isolated were four copies of *cox1 *from *Crypthecodinium cohnii*, each of which was found to occur in a unique genomic context [15]. Southern blots demonstrated multiple different copies of this gene that varied in abundance, suggesting the *C. cohnii *mitochondrial genome is not as streamlined as in apicomplexans. Subsequently, *cob *and *cox3 *have been found as well, and multiple, sometimes fragmented copies of these genes have now been reported from diverse dinoflagellates (*Gonyaulax polyedra*, *Pfiesteria piscicida*, *Alexandrium catenella*) [16-18]. Most unexpected, however, was the demonstration that protein-coding transcripts are heavily edited at the RNA level in diverse dinoflagellates [18,19], unlike the case in either apicomplexans or ciliates.

To gain greater insight into the nature of dinoflagellate mitochondrial genomes, we have generated a large body of mitochondrial genomic and transcriptional data for two distantly related dinoflagellate species, *C. cohnii *and *Karlodinium micrum*. These data encompass more than 30 mtDNA fragments totaling > 42 kb, and more than 50 mitochondrial transcripts. This new information highlights several novel features of the organization and expression of the dinoflagellate mitochondrial genome, and concurrent studies in two additional distantly related dinoflagellates, *Amphidinium carterae *[20] and *Oxyrrhis marina *[21], corroborate a number of our findings. Together, these data reinforce the conclusion that the dinoflagellate mitochondrial genome has been substantially reorganized since the divergence of dinoflagellates and apicomplexans from a common ancestor.

## Results

### Genomic sequence reveals a complex mitochondrial genome

#### *Crypthecodinium cohnii*

**Figure 1:**
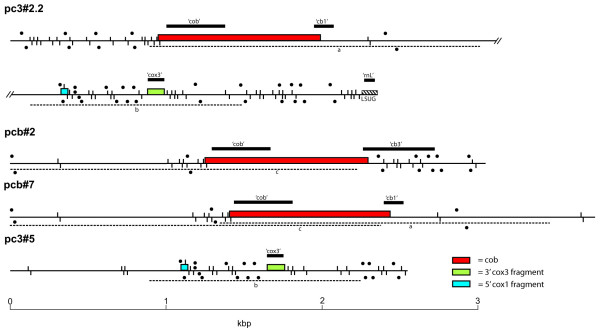
**Schematic of *C. cohnii *mtDNA fragments**. Mitochondrial sequences are drawn to scale, with coding sequence on either the forward or reverse strand indicated above or below the line, respectively. Colored blocks indicate protein-coding genes and hatched boxes denote rRNA genes. Coding sequence is identified by sequence similarity to gene homologues irrespective of standard start and stop codons. Common sequence between fragments (> 99% identity) is indicated by horizontal dashed lines and matching lowercase letters. Black boxes indicate locations and sizes of Southern blot probes. Large inverted repeats (> 9) are indicated by black dot pairs above and below each sequence, and short proximal inverted repeats (> 6) are indicated by paired vertical dashes. Minor differences of inverted repeat distribution between common sequence (dashed lines) are due to the minor sequence differences.

Previously reported *C. cohnii cox1 *sequences indicated multiple copies of the gene with different flanking sequences [15]. To test if this genomic complexity extends to other *C. cohnii *mitochondrial genes, we sequenced multiple genomic clones containing *cob *and/or *cox3*. A library of *Eco*RI restriction fragments constructed from a fraction enriched in mtDNA was screened using a *C. cohnii cob *gene probe, obtained by PCR. This screen recovered a *cob *clone linked to a 57-bp *cox3 *fragment, which itself was used to probe for *cox3*-containing clones. In total, 14 clones were characterized (11 *cob*, two *cox3 *and one containing both), ranging in size from 2.5 kb to 5.4 kb (eight clones were 3.7 kb long). End sequencing and restriction mapping identified six unique *cob*-containing clones, and three unique *cox3*-containing clones. Four clones were completely sequenced (Figure 1).

The largest clone, pc3#2.2 (5.4 kb), contains a complete or nearly complete *cob *gene (see below), followed by three other identifiable sequences: a 49-bp stretch identical to a sequence previously found in a *cox1*-containing clone [15]; a 113-bp *cox3 *segment; and a 99-bp large subunit (LSU) rRNA sequence corresponding to mitochondrial LSUG in apicomplexans [14]. Two additional *cob *clones were sequenced, pcb#7 (3.7 kb) and pcb#2 (3.2 kb). Both encode *cob*, but with different flanking sequences than in pc3#2.2. pcb#2 contains unique 3' sequence immediately after the *cob *repeat, whereas pcb#7 contains additional common sequence with pc3#2.2 for ~1 kb before unique sequence occurs (Figure 1). Amongst these clones, we observed two different 5'-flanking sequences and three different 3'-flanking sequences (Figure 1). This arrangement recapitulates the organization of *cox1 *in *C. cohnii *mtDNA [15], i.e., a central repeat (1072 bp) containing most of the *cob *ORF) flanked by different arrays of unique upstream and downstream sequences. Partial sequencing of the remaining clones revealed an additional unique 5'-flanking sequence (in pcb#8) and one additional unique 3'-flanking sequence (in pcb#4 and pcb#9) in the immediate vicinity of the *cob *ORF (data not shown).

Of the three *cob*-containing clones described above, only pcb#2 encodes a complete cytochrome *b *(Cob) protein (see below). pc3#2.2 and pcb#7 share an alternative 3' sequence that predicts a Cob C-terminal sequence lacking 24 amino acid residues compared with the pcb#2-predicted Cob as well as the corresponding *Plasmodium falciparum *Cob. This suggests that the pc3#2.2 and pcb#7 Cob ORFs represent pseudogenes. Variable 3' coding sequences were also seen previously for *C. cohnii cox1*, with some coding sequences also truncated compared to other dinoflagellate sequences [15].

One *cox3*-containing clone (pc3#5) was also sequenced, but it was found not to encode an intact *cox3 *gene. Instead, this clone encoded 1339 bp identical in sequence to the portion of pc3#2.2 that included the 113-bp *cox3 *segment and the 49-bp *cox1 *sequence (Figure 1). This clone was also flanked by unique sequences, providing further evidence that mitochondrial genes occur in multiple genomic contexts in *C. cohnii*.

**Figure 2:**
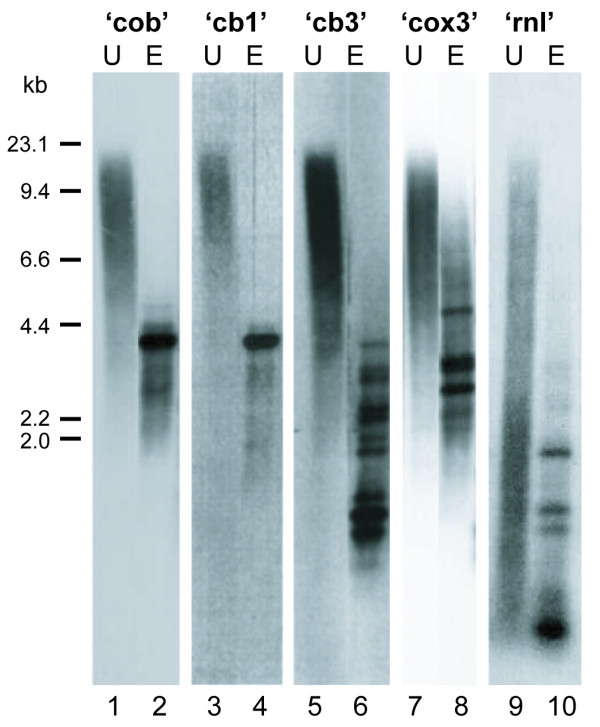
**Southern blot analysis of *C. cohnii *mtDNA with ^32^P-labelled probes specific for mitochondrial gene and flanking regions**. A fraction enriched in mtDNA was either untreated ('U') or *Eco*RI hydrolysed ('E') and the products separated by gel electrophoresis. Blots were hybridized with probes specific for *cob*, *cob*-flanking sequences ('cb1' and 'cb3'), *cox3 *or LSUG ('rnl') (see Figure 1 for probe locations). Size markers are indicated to the left in kb pairs.

To further investigate the arrangements and relative numbers of mtDNA elements, Southern hybridization analysis was performed using region-specific probes. As shown in Figure 1, probes were generated specific to: the *cob *coding sequence ('cob'); two *cob *3'-flanking regions ('cb1', specific to pc3#2.2 and pcb#7; and 'cb3', specific to pcb#2); the *cox3 *sequence ('cox3'); and the rRNA sequence LSUG ('rnl'). These probes were hybridized against a mtDNA-enriched fraction hydrolyzed by *Eco*RI. With the 'cob' probe, a strong signal was detected at 3.7 kb and weaker signals at 4.8, 4.5, 3.5, and 3.0 kb (Figure 2). This result is consistent with dominant *Eco*RI clones being 3.7 kb, and with multiple genomic contexts for *cob*. Probing with 3' flanking sequence 'cb1' revealed a similar banding pattern to that generated by the 'cob' probe, indicating that this region is typically contiguous with the *cob *coding sequence. Probing with 'cb3' presented a very different profile, with 10 bands ranging in size from 3.7 to 0.5 kb and of varying intensity (Figure 2). The cb3 sequence evidently occurs in numerous *Eco*RI fragments, some without *cob*. Probing with 'cox3' and 'rnl' also revealed multiple bands with varying intensity (Figure 2), again indicating that these mtDNA elements are present in several different genomic arrangements. Together these Southern data verify the existence of multiple copies of *C. cohnii *mtDNA elements occurring in different contexts, and indicate that up to 10 different arrangements occur for some of these elements.

#### *Karlodinium micrum*

Putative mitochondrial genes were identified from a survey of 16544 *K. micrum *expressed sequence tag (EST) sequences assembled into 11903 unique clusters [22]. Oligoadenylation of mitochondrial gene transcripts is known from other organisms [23,24], and this also appears to be the case in dinoflagellates as the poly(A)-dependent *K. micrum *survey also contained many cDNAs for mitochondrial genes. Mitochondrial sequences were identified by homology to genes in other systems, and all such cDNAs were fully sequenced. Using this strategy we identified sequences representing the three protein-encoding genes found in *C. cohnii*: *cox1 *(1 cDNA), *cob *(11 cDNAs) and *cox3 *(9 cDNAs). The average A+T content of these sequences was 69% (compared to 49% for nuclear genes, calculated from all 11903 *K. micrum *clusters), consistent with their being encoded in the mitochondrion. We found no other mitochondrial protein-coding sequences exhibiting the strong A+T biases suggestive of an origin from mtDNA (*cox2 *coding sequence, for example, which is typically encoded in mitochondria but is known to have been transferred to the nucleus in dinoflagellates [25], contains 47% A+T). Several short cDNA sequences, however, with high similarity to the fragmented apicomplexan mitochondrial rRNAs [14] (see also GenBank acc. no. M76611 for updated annotation) were identified. These correspond to apicomplexan LSU rRNA fragments LSUA, RNA2, LSUE, LSUG and RNA10 (3, 1, 3, 1, and 9 cDNAs, respectively), small subunit (SSU) rRNA fragment RNA8 (9 cDNAs), and an RNA (RNA7, 7 cDNAs) that has yet to be assigned to either the LSU or SSU rRNA. While these sequences have a lesser A+T bias (56%) compared with the mitochondrial protein-encoding sequences, the high similarity of these sequences to their apicomplexan counterparts (see below), and known oligoadenylation of these transcripts in apicomplexans [23,24], strongly implicates these sequences as additional elements of the *K. micrum *mtDNA.

**Figure 3:**
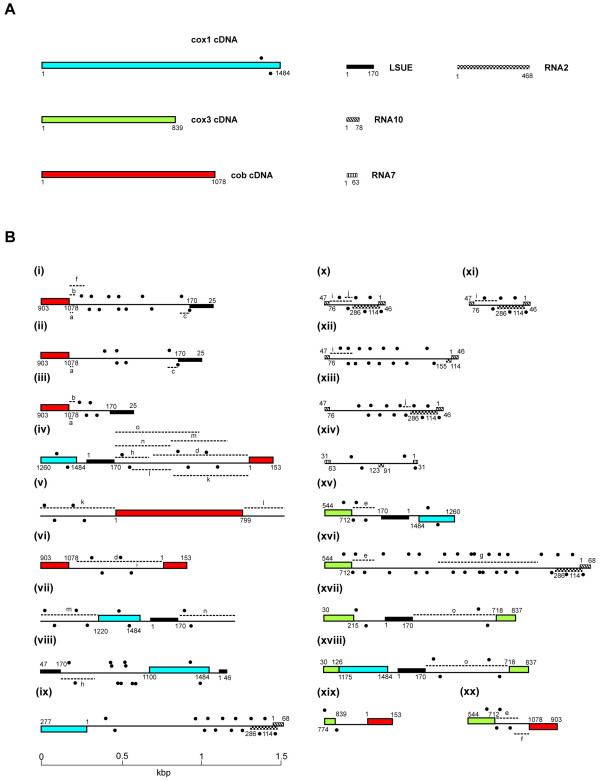
**Schematic of *K. micrum *mitochondrial cDNAs (A) and 20 mtDNA fragments generated by PCR (B)**. Gene sequences in (A) correspond to the longest cDNA data generated for each gene (see also Figure 4). Mitochondrial sequences are drawn to scale, with coding sequence in (B) on either the forward or reverse strand indicated above or below the line, respectively. Colored blocks indicate protein-coding genes, textured black boxes indicate rRNA genes. cDNA lengths (in nucleotides (nt)) are indicated in (A), and corresponding nucleotide matches in PCR fragments are accordingly indicated in (B). Common intergenic sequences (> 99% identity) between PCR fragments are indicated by dashed lines and matching lowercase letters. The letter 'g' indicates matching sequence to unidentified cDNA EF443049. Inverted repeats are indicated by black dot pairs above and below each sequence.

With these 10 mtDNA tags, we used PCR to generate genomic sequences corresponding to each gene and regions linking them, with the aim of assembling large portions of *K. micrum *mtDNA sequence. Intergenic sequence recovered by this approach was used to provide further priming sites to extend the sampling of *K. micrum *mtDNA. In addition to amplification of individual genes, a total of 20 distinct gene linkage products were generated and fully sequenced (Figure 3B). This analysis yielded a sequence in which mitochondrial genes were linked to one another in many different contexts. Gene fragments were also common, as were mtDNAs with three or four distinct fragments or tandem repeats (Figure 3B). In total, *cob *sequences were found in at least six mutually exclusive linkages, *cox3 *in five, *cox1 *in four, LSUE in nine, RNA10 in six, RNA2 in five and RNA7 in one. Additionally, two large cDNAs (GenBank accession EF443051, 5 854 bp; and EF443052, 2153 bp) provided further evidence of multiple copies of mitochondrial genes and gene fragments linked in novel arrangements. EF443051, for example, contains the LSUG coding sequence, a second partial LSUG unit within a 170-bp repeat, the LSUA sequence, the RNA8 sequence, and an internal fragment of the *cox1 *gene (73 bp). These cDNAs also indicate that polycistronic transcription occurs in dinoflagellate mitochondria.

Intergenic sequences from the PCR clones were examined for additional coding elements by comparison to publicly available databases, specifically searching against *K. micrum *ESTs as well as comparing the intergenic regions to one another. No identifiable genes were found, but one cDNA sequence (GenBank accession EF443049) was represented in one mtDNA clone, implicating this sequence as an additional transcriptional unit of the mitochondrial genome (Figure 3B, xvi). Comparison of intergenic sequences to one another revealed numerous dispersed repeated sequences with either 100% or very high degrees of identity (Figure 3B, dashed lines). Overall, data from *K. micrum *are consistent with those from *C. cohnii*, both pointing to a complex genome organization evidently underpinned by a high level of recombination within dinoflagellate mitochondria.

### Inverted repeats in mtDNA

Previous analysis of *C. cohnii cox1 *identified many short inverted repeats in flanking, non-coding sequences [15]. We have applied a similar analysis to the *C. cohnii cob*- and *cox3*-containing sequences, as well as the *K. micrum *mtDNA data, and find a very similar pattern of repeat features, although we also note some differences between the two taxa. Within the *C. cohnii *sequences, we screened for inverted repeats of different length and distance between them, and found two distinct but prevalent classes of this element type. The first class is similar to those previously described [15], and consists of very closely spaced, small inverted repeats (> 6 nucleotides and no more than 5 nucleotides apart). These inverted repeats occur almost exclusively within non-coding sequence, with the only exceptions being at the very extremities of genes (Figure 1, vertical dashes). A second class of inverted repeats consists of longer repeat elements (> 9 nucleotides) no more than 50 nucleotides apart. Such inverted repeats are also prevalent in *C. cohnii *mtDNA, and are almost exclusively features of the non-coding sequences (Figure 1, small circles).

Analysis of *K. micrum *mtDNA showed that inverted repeats are also a feature of intergenic sequences; however, in this case only the larger class of inverted repeats was found, with none of the smaller, closely spaced inverted repeats occurring in any of the mtDNA sequences (Figure 3). Again these repeats are almost exclusively located within intergenic regions, with genic inverted repeats only occasionally present, within gene extremities. No equivalent inverted repeats were found in a random sample of 10 *K. micrum *nucleus-encoded gene sequences (10630 nucleotides total). The sequences of repeated elements in both *C. cohnii *and *K. micrum *are consistent with secondary structures such as stem loops and hairpins, and in both cases the repeated elements that could form such stem structures are typically G+C rich, in spite of the A+T bias of these organelle genomes. The inverted repeats described here are also distinct from secondary structural elements of the rRNAs (see below) that typically consist of imperfect inverted repeats. Densely packed inverted repeats, primarily in intergenic regions, was also recently described from *A. carterae *mtDNA [20]. In this case, imperfect inverted repeats were predicted to form stems of 50–150 nucleotides, with AT-rich loops of ~10–30 nucleotides. While inverted repeats therefore appear to be a consistent feature of dinoflagellate mitochondrial genomes, the elaboration of these elements is variable between taxa, with shorter repeats only present in *C. cohnii*.

### Mitochondrial gene transcripts lack stop and start codons

Extensive substitutional RNA editing of transcripts occurs in dinoflagellate mitochondria, so exactly where an open reading frame begins and ends can only be tentatively inferred from genomic DNA. Accordingly we used *K. micrum *cDNAs, and publicly available mRNA sequences from several other dinoflagellates, to identify the ends of all three protein-coding genes.

#### Absence of stop codons

**Figure 4:**
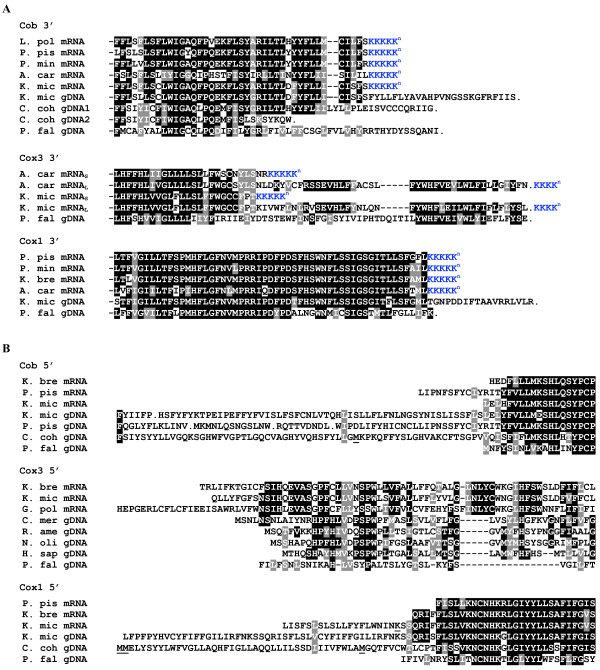
**Absence of conventional stop and start codons represented in protein alignments of dinoflagellate Cob, Cox3 and Cox1**. Predicted amino acid sequence termini represent (A) 3' and (B) 5' sequences from cDNA and gDNA. Blue sequence indicates conceptual translation of 3' oligo(A) tract of mRNAs. Identical and similar residues are indicated by black or grey backgrounds, respectively. Inferred differences between cDNA and gDNA sequences of the same taxa correspond to RNA editing changes. Only longer *cox3 *mRNAs (mRNA_L_) encode an in-frame stop codon, generated by oligoadenylation following a terminal U. The 5' sequence termini represent either the limit of reverse transcription of mRNAs, or inferred translations of 5' genomic coding sequence (gDNA). Cob 3' sequence 'C. coh gDNA1' corresponds to clone pcb#2, while 'C. coh gDNA2' corresponds to clones pc3#2.2 and pcb#7. Underlined K in 'K. mic mRNA' (B, Cox1 5') indicates the site of a 10-nt deletion relative to 'K. mic gDNA'. Underlined Ms (B, Cob 5' and Cox1 5') indicate possible initiation codons found in-frame, but upstream of conserved sequence. Non-dinoflagellate homologues included for comparison of protein termini are: P. fal, *Plasmodium falciparum *M76611; C. mer, *Cyanidioschyzon merolae*, BAA34657; R. ame, *Reclinomonas americana*, AAD11871; N. oli, *Nephroselmis olivacea*, AAF03208; H. sap, *Homo sapiens*, AAZ02899. Dinoflagellate taxa and accession numbers: K. mic, *Karlodinium micrum*, this study; C. coh, *Crypthecodinium cohnii*, this study; L. pol, *Lingulodinium polyedrum*, CD810189, CD810189; G. pol, *Gonyaulax polyedra*, AF142470; P. pis, *Pfiesteria piscicida*, AF357518, AF463413, AF357518, AF357521; K. bre, *Karenia brevis*, CO062170, CO065693, CO062289, CO060561; A. car, *Amphidinium carterae*, CF064846, CF065669, CF064811, CF067165; P. mic, *Prorocentrum minimum*, AY030285, AF463415.

Oligoadenylation of transcripts apparently occurs upstream of any canonical stop codon in all protein-encoding transcripts analyzed, and for only one gene does oligoadenylation create an in-frame canonical stop codon. This lack of encoded stop codons applies to transcripts for *cob, cox3 *and *cox1 *represented from multiple species. All 11 *cob *transcripts from *K. micrum *are oligoadenylated at the same point, which corresponds to the expected C-terminus of Cob homologues (Figure 4), but does not include an in-frame stop. The 3' ends of transcripts from four other dinoflagellates (*P. piscicida, Prorocentrum minimum*, *G. polyedra, A. carterae*) are oligoadenylated at precisely the same position (Figure 4). For *cox1*, the mRNA sequences from four taxa (*P. minimum, P. piscicida, A. carterae*, and *Karenia brevis*) are all oligoadenylated at the same position, where the protein sequence is predicted to terminate (Figure 4); once again, none of these encode a stop codon.

The *K. micrum cox3 *cDNAs present an even more interesting situation. Five of nine cDNAs are oligoadenylated approximately 40 codons upstream of the predicted C-terminus, and without an in-frame stop codon (Figure 4). However, another four cDNAs are oligoadenylated a further 129 nucleotides downstream; these cDNAs encode amino acid sequence with high similarity to the C-terminus of Cox3. In this case, oligoadenylation follows a U residue creating an in-frame UAA stop codon. The generation of an in-frame stop codon concomitant with oligoadenylation is also apparent in *Amphidinium cox3 *mRNA; however, as in *K. micrum*, other *cox3 Amphidinium *transcripts are oligoadenylated prematurely, within a few bases of the premature oligoadenylation site in *K. micrum *cDNAs (Figure 4). Alternative oligoadenylation sites have also been reported for *cox3 *transcripts in the dinoflagellate *G. polyedra *[16].

A potential alternative stop codon was sought among these transcript data by looking for a codon that occurs exclusively in the 3' region of these coding sequences. However, no such candidate codon could be identified either within or between the taxa surveyed, nor is there any evidence for use of a non-standard genetic code (with the possible exception of start codons, see below). Moreover, oligoadenylation consistently occurred at the position where the protein sequence is expected to terminate, leaving little or no apparent untranslated region (UTR).

#### Alternative start codons

Dependence on a standard ATG start codon also is apparently relaxed in dinoflagellate mitochondria. From multiple dinoflagellate species mRNAs for the three protein-coding genes extend beyond conserved N-termini, suggesting these transcripts are likely to be full length, but all lack a plausible N-terminal AUG (Figure 4). Existing genomic sequences corroborate the lack of initiating ATGs.

Transcript data for *cox3 *from three species (*K. brevis, K. micrum *and *G. polyedra*) and *cox1 *from *K. micrum *are all apparently full length based on protein alignments and all lack an AUG in the terminal region (Figure 4). The corresponding genomic region upstream of *K. micrum cox1 *does not contain an in-frame ATG until 615 nucleotides upstream of the conserved sequence, and 11 stop codons fall between them, supporting the likely absence of an ATG from this gene. Genomic sequences for *C. cohnii cox1*, however, do contain an in-frame ATG ~13 codons upstream of N-terminal sequence conservation seen among dinoflagellates. While it is possible that this particular ATG serves as the initiator codon in this taxon, the lack of any sequence conservation with the corresponding *K. micrum *sequence within this 13-residue stretch (Figure 4) suggests that this might also represent a chance ATG within the 5' UTR.

*K. micrum cob *mRNAs do encode an AUG close to the site where sequence conservation with other Cob proteins begins, but on close inspection there is conserved sequence upstream of this codon (Figure 4). Further, *cob *from the early-diverging member of the dinoflagellates, *Oxyrrhis marina*, lacks this AUG or any other upstream of this region [21]. In mRNAs of all other available species (*K. micrum*, *K. brevis*, and *P. piscicida*) there is strong conservation of the four predicted amino acid residues upstream of this ATG (F, V/L, L, L), further suggesting that translation likely initiates upstream of it (Figure 4). The conservative change of this second residue, V to L, among dinoflagellate taxa (and V to I in the genomic sequence for *C. cohnii*) supports the inference that this region likely represents protein-coding sequence rather than UTR. Some conservation of this sequence with *Plasmodium *Cob is also apparent (Figure 4). None of the four apparently full-length *K. micrum cob *genomic sequences encodes an additional ATG codon between this region of conservation and the next in-frame stop codon (Figure 4), and the same situation is seen in a *P. piscicida cob *sequence. The *C. cohnii *genomic sequences are the only cases to date where potential ATG codons do occur in this upstream sequence (Figure 4). However, two of these occur well upstream of any 5'-sequence conservation among dinoflagellates, and would represent unusually long (5'-extended) and divergent Cob proteins in these cases (Figure 4).

### Trans-splicing of *cox3*

Included among the *K. micrum cox3 *cDNAs were four inferred to be full length (839 nucleotides) based on protein alignments (Figure 4), and five inferred to be prematurely oligoadenylated at nucleotide 712. Despite the fact that the longer cDNA is likely the functional *cox3 *mRNA, a genomic copy corresponding to it could not be amplified from genomic DNA using multiple primer combinations (all of which successfully amplified the corresponding fragments in RT-PCRs; data not shown). The longest product obtained from genomic DNA corresponded to nucleotides 50–712 of the full-length *cox3 *sequence. Six genomic fragments containing *cox3 *sequence were obtained by amplifying between genes, and these suggest that the gene is fragmented in the genome (Figure 3B, xv, xvi, xvii, xviii, xix and xx). Notably, three unique *cox3 *genomic sequences are truncated at nucleotide 712, precisely where the short *cox3 *transcripts are oligoadenylated (Figure 3B, xv, xvi and xx). Immediately downstream is a stop codon, and subsequently no further sequence similarity to *cox3*. Similarly, the only genomic sequences found to encode the 3' end of the long transcript are 5'-truncated at nucleotide 718, with sequence unrelated to *cox3 *upstream of this point (Figure 3B, xvii and xviii). Taken together, these data suggest that the long *cox3 *transcript is the product of trans-splicing, where nucleotides 1–712 are joined to nucleotides 718–839 arising from two different genomic fragments. The intervening five nucleotides (713–717) are all A residues in the full-length *cox3 *transcript, suggesting that trans-splicing occurs within the oligo(A) tail of the upstream transcript.

### Mitochondrial rRNAs are fragmented in a similar pattern as in apicomplexans

**Figure 5:**
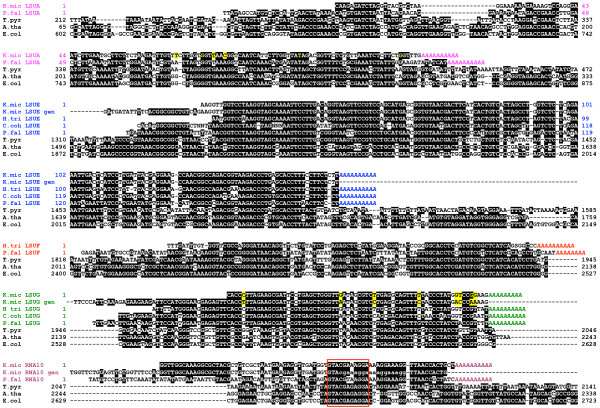
**Dinoflagellate LSU rRNA sequences aligned to those of their fragmented apicomplexan counterparts**. Intact LSU rRNAs from the mitochondrion of a ciliate and plant and from a bacterium are included in the alignment. Color groups indicate distinct rRNA cDNAs with oligoadenylation shown in italics. *K. micrum *genomic sequence (gen) is included for LSUE, LSUG and RNA10 (lowercase sequence denotes primer sites used for RNA10 gen). Yellow highlights differences between *K. micrum *genomic and cDNA sequences. Red box indicates the conserved domain of the sarcin/ricin loop represented in RNA10 sequences. K.mic, *Karlodinium micrum*; H.tri, *Heterocapsa triquetra*; C.coh, *Crypthecodinium cohnii*; P.fal, *Plasmodium falciparum *M76611; A.tha, *Arabidopsis thaliana*, Y08501; T.pyr, *Tetrahymena pyriformis*, M58010; E.col, *Escherichia coli*, D12649.

SSU and LSU rRNAs are encoded in all characterized mtDNAs; however, until recently [17] no mitochondrial rRNA sequences had been described from dinoflagellates. In this study we have identified several discrete, short sequences with strong similarity to components of the highly fragmented rRNAs of apicomplexans [14] (GenBank acc. no. M76611). From *K. micrum*, we obtained cDNA sequences representing five LSU rRNA fragments (LSUA, RNA2, LSUE, LSUG, and RNA10), one SSU rRNA fragment (RNA8), and one unassigned rRNA fragment (RNA7), all of which correspond to known transcriptional units of the *Plasmodium *mitochondrial genome. We also identified an additional LSU rRNA fragment, LSUF, as well as LSUE and LSUG, from an EST survey we previously conducted in *Heterocapsa triquetra *[26]. Alignment of LSUA, LSUE, LSUF, LSUG and RNA10 to their *Plasmodium *LSU homologues is shown in Figure 5. SSU rRNA fragment RNA8 and unassigned fragment RNA7 share 66% and 74% sequence identity to *Plasmodium *homologues, respectively. For each fragment, multiple cDNAs were sequenced (with the exception of RNA2 and LSUG), and oligoadenylation was found to occur at a consistent site (Figure 5). Although these cDNAs are all relatively short, the 5' ends could not be definitively determined from these cDNAs because the 5'-lengths were variable. Further, genomic copies (where they are known) encoded conserved sequence upstream of the 5' ends of cDNAs of LSUE and LSUG (Figure 5).

For *C. cohnii*, the LSUG sequence identified on *Eco*RI clone pc3#2.2 was analyzed by 3' RACE and the site of oligoadenylation was shown to be identical to that in the corresponding *K. micrum *and *H. triquetra *cDNAs (Figure 5). Northern analysis of *C. cohnii *RNA showed a single LSUG-positive band at ~108 nucleotides [27]. This size corresponds well with the limit of conservation among LSU rRNA sequences, as well as the size of the *Plasmodium *LSUG. *C. cohnii *LSUE was also amplified and the ends determined by 5'-cDNA sequencing and 3' RACE (Figure 5). Northern hybridization against mitochondrial RNA confirmed the presence of an ~200 nucleotide RNA species [27].

**Figure 6:**
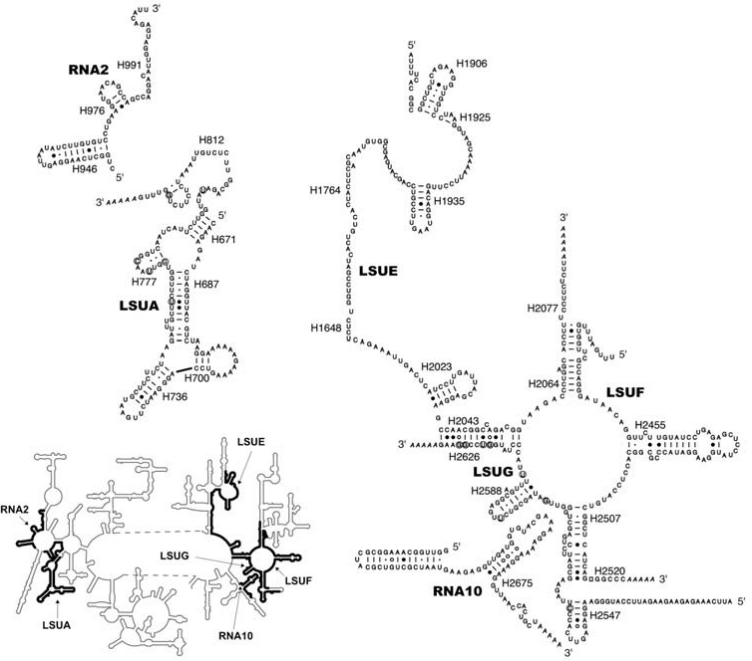
**Predicted secondary structures of dinoflagellate mitochondrial LSU rRNA fragments**. RNA sequences were deduced from RNA and DNA sequences, and structures were modelled on the secondary structure of *E. coli *LSU rRNA. Fragments correspond to *K. micrum *RNA2, LSUA, LSUE, LSUG and RNA10, and *H. triquetra *LSUF. Note that the potential hairpin at the 5' end of RNA10 does not have a counterpart in *E. coli *LSU RNA. Only a portion of the RNA2 cDNA sequence is shown; also, the actual 5' terminus of LSUA (and LSUF) likely extends past the sequence shown. Positions of the dinoflagellate fragments are mapped onto the full *E. coli *LSU rRNA structure, inset. Putative Watson-Crick and wobble base pairs are indicated by lines and dots respectively, GoA pairs by open circles, and non-canonical pairs by closed circles. Positions enclosed by a circle are editing sites, with the post-edited nucleotide shown. Oligoadenylation is indicated by italics. Helices are numbered according to the *E. coli *23S rRNA structure [61].

The oligoadenylation sites for mitochondrial rRNA fragments are identical among dinoflagellates, and either identical or within a few nucleotides of those observed in *Plasmodium *(Figure 5). The 5' ends of these sequences, whether defined experimentally (LSUE and LSUG from *C. cohnii*) or by sequence conservation, are also very similar to those of their *Plasmodium *counterparts. The only possible exception is *K. micrum *RNA2, where the sole cDNA obtained contained substantial upstream (305 nucleotides) and downstream (79 nucleotides) sequence compared to the region with similarity to *Plasmodium *RNA2. However it is possible that this cDNA represents an unprocessed precursor, and accordingly further work is required to substantiate the size of this putative rRNA fragment. Secondary structure predictions for dinoflagellate sequences LSUA, LSUE, LSUF, LSUG, RNA10 and putative RNA2 (limited to the region of similarity to the *Plasmodium *RNA2) all indicate that the expected folding and intermolecular base pairings occur (Figure 6), and these fragments are likely to contribute to a viable reconstituted LSU rRNA, as for *Plasmodium*.

### RNA editing

#### Protein-coding genes

RNA editing has been described for *cox1, cob *and *cox3 *transcripts from diverse dinoflagellates, including the *cob *mRNA of *K. micrum *[18-20]. Comparison of *K. micrum *cDNA and corresponding mtDNA sequences for the three genes identified here confirms this conclusion for transcripts of *cob*, and further shows that *cox1 *and *cox3 *transcripts are also edited. The average density of editing of the *cox1 *transcripts is one substitution per 36 nucleotides and this value is consistent with other studies in different species [18,19]. By contrast, editing in *cox3 *transcripts is over twice as dense, at one substitution per 17 nucleotides, making *cox3 *the most heavily edited gene transcript in dinoflagellates. Editing of *cob *mRNA lies in between these extremes, at one substitution per 25 nucleotides.

In the case of *cox1 *transcripts, four types of substitutional changes were detected at 42 sites. Of these, 48% were A to G substitutions, followed by U to C (21%) and smaller proportions of C to U and G to C edits (17% and 14%, respectively). This observation is consistent with *cox1 *mRNA editing occurring in other species, where most (80%) of the reported changes are A to G and U to C substitutions [18,19]. So far, G to C changes have only been observed in mtDNA-encoded mRNAs of dinoflagellates, whereas A to G changes have only been reported in nucleus-encoded mRNAs. *cox3 *mRNA editing types are generally consistent with those observed in *cox1 *and *cob *mRNAs. Five types of substitutional changes were observed at 50 sites, of which 42% were A to G changes, followed by C to U and U to C edits (28% and 22% respectively), as well as three G to A edits (6%) and a single G to C edit (2%). For both *cox1 *and *cox3 *mRNAs, the majority of substitutions occur at the first or second positions of affected codons (88% and 96%, respectively), and over 90% of editing events result in a change in predicted amino acid. In *K. micrum cox3 *mRNA (and *cox1 *and *cob *mRNAs of other dinoflagellates [18,19]), editing also removes a UAG codon, which is typically a stop codon but is apparently unassigned in dinoflagellates.

Analysis of the 20 cDNAs corresponding to *cox3 *and *cob *offers further insight into the process of RNA editing in dinoflagellates. Despite overall uniformity of transcript editing, some cDNAs exhibit pre-edited states. *K. micrum cox3 *and *cob *contain 50 and 44 editing sites, respectively, with the cDNAs analyzed here representing in total 343 and 231 potential editing events, respectively. However at nine of these sites in the *cox3 *cDNAs, and five in the *cob *cDNAs, the pre-edited nucleotide occurs, indicating 2.6% and 2.2% 'non-edits', respectively. These 'non-edits' were present in only a few cDNAs (two and three for *cob *and *cox3*, respectively), suggesting that the great majority of cDNAs represent mature transcripts. The pre-edited sites are scattered throughout the transcripts where they are found, occur between other edited sites, and in no obvious order in any sequence. These pre-edited sites may indicate editing failures, in which case such transcripts could give rise to defective translation products. Alternatively, they may represent editing intermediates. If the latter is the case these data suggest that editing does not occur in a linear sequence along each transcript. Pre-edited mitochondrial cDNAs have also recently been found in *A. carterae *mtDNA [20].

#### rRNA transcripts

Comparisons of rRNA cDNAs to genomic sequences are constrained by the smaller sizes of these sequences (for example 63 nucleotides for RNA7), in particular where PCR has been used to amplify genomic sequence a greater portion of this sequence represented primer binding sites and therefore cannot be used in such a comparison. Nevertheless, from the available data, there is no evidence of editing of RNA8, RNA10 or RNA7. For LSUE, complete genomic sequence (170 nucleotides) was available from the internal regions of five PCR fragments, with the majority of the sequence available from a further four PCR products using LSUE primers. These sequences were identical to the cDNAs except for three consecutive nucleotides that were absent in two of the three LSUE cDNAs obtained from the EST survey. To test this anomaly, a further five cDNAs were independently generated, and these all contained the three nucleotides, and therefore were identical to genomic LSUE sequences and to one of the original EST sequences. These results suggest that the three-nucleotide deletions seen in two cDNAs represent a rare artifact, likely generated during reverse transcription, and that *K. micrum *LSUE is likely also not edited.

There was, however, evidence of substitutional editing for LSUA and LSUG. In both cases genomic copies of these sequences differed from transcripts: in LSUG at eight positions and in LSUA at six positions (Figures 5 and 6). Consistent with the protein-coding genes, these substitutions consist mainly of A to G (36%), C to U (43%) and U to C (14%) substitutions, with one case of C to G. Given that dinoflagellate mitochondrial genes occur in multiple copies, recovery of further, independently isolated copies of these genes will be required to substantiate these inferences of rRNA editing. Evidence for rRNA editing has also recently been reported with the dinoflagellate *A. catenella*, where two inferred editing events were identified for the 'LSUE-like' rRNA [17].

## Discussion

Prior to this study our view of the dinoflagellate mitochondrial genome was gleaned from relatively sparse molecular data obtained from several diverse dinoflagellate taxa. These data nevertheless provided a tantalizing view of a mitochondrial genome displaying several eccentricities. Coding sequences for entire or partial versions of *cox1*, *cob *or *cox3 *have been shown to occur in multiple copies and in different genomic contexts in *C. cohnii *[15,27], *G. polyedra *[16], *P. piscicida *[28], and *A. catenella *[17]. These data paint a picture of dinoflagellate genomes in sharp contrast to the minimalist 6 kb apicomplexan mtDNA, which encodes single copies of these genes, tightly packed together [13]. Similarly, extensive RNA editing has been described in mRNAs from diverse dinoflagellates [18-20], a process that does not occur in apicomplexans. In this study we have generated a much more comprehensive body of mitochondrial genomic and transcript data for two dinoflagellate species, *C. cohnii *and *K. micrum*, and these data are bolstered by a concurrent mitochondrial genomic study of the dinoflagellate *A. carterae *[20]. Together, these results reinforce the view that the dinoflagellate mitochondrial genome has diverged radically in form from that of apicomplexans, despite the persistence of some intriguing similarities.

### Mitochondrial genome content and form

Compared to the complement of 43 to 52 genes in the mitochondrial genome of ciliates [12], the most basal member of the phylum Alveolata, the very low information content of apicomplexan mtDNA (three protein-encoding genes – *cox1, cox3 *and *cob *– and ~23 short transcription units that encode the functional SSU and LSU rRNAs) clearly shows that there has been considerable mitochondrial gene loss and/or relocation to the nucleus during alveolate evolution. We infer that much, if not all, of this gene relocation must have occurred prior to the last common ancestor of dinoflagellates and apicomplexans. In EST surveys, we have only identified the same three protein-coding genes (*cox1, cox3 *and *cob*); moreover, we found no other mitochondrial ORFs of known function in > 28 kb and > 14 kb of mtDNA sequence from *K. micrum *and *C. cohnii*, respectively. These findings are consistent with the previous demonstration that *cox2*, an otherwise nearly ubiquitous component of mitochondrial genomes, has been relocated to the nucleus in both apicomplexans and dinoflagellates [25,29]. The only additional genes we identified are ones representing the mitochondrial SSU and LSU rRNAs, which together with *cox1 *and *cob *are universally present in mtDNA. No tRNA genes have been found linked to mtDNA sequences, similar to apicomplexans, where tRNAs are apparently imported into mitochondria from the cytoplasm [13].

Dinoflagellates and apicomplexans also share the characteristic of highly fragmented SSU and LSU rRNAs. Fragmentation of mitochondrial rRNA genes has been documented in the mitochondrial genomes of several eukaryotes, including ciliates [30,31], several green algae [8,32-36] and a fungus [37]. The degree of fragmentation in apicomplexan mitochondrial rRNA is more extreme than in these other cases, with 23 fragments for the SSU and LSU rRNAs reported to date, coding regions for which are rearranged and interspersed with other genes in the genome [14]. From within three disparate dinoflagellate taxa we have identified eight rRNA fragments similar to fragments in *P*. *falciparum*, and three of these rRNA species have also recently been reported from two further taxa, *A. catenella *and *O. marina *[17,21]. The dinoflagellate rRNA fragments mostly appear to correspond to their *Plasmodium *counterparts in length and sequence termini, suggesting that a stable level and pattern of fragmentation has been inherited from the common ancestor of dinoflagellates and apicomplexans. Given that ciliate mitochondrial rRNAs are comparatively intact (encoding bipartite SSU and LSU rRNAs, and with only the fragmented LSU rRNA gene rearranged; see [12]), the extreme fragmentation in dinoflagellates and apicomplexans must have occurred since their divergence from ciliates.

Despite a similar gene content the arrangement of dinoflagellate and apicomplexan mitochondrial genomes is radically different. Where the apicomplexan genome is relatively simple and compact, the dinoflagellate mitochondrial genome is complex, with multiple copies of each gene imbedded within different genomic contexts. Gene fragments and non-coding regions are also repeated, altogether suggesting a great deal of recombination in the genome, which is also consistent with the lack of sequence divergence among the multiple copies of these elements. Shotgun sequence data recently published for the *A. carterae *mitochondrion corroborate this picture of a recombining complex genome, and further suggest that the majority of the mitochondrial genome (~85%) might be non-coding [20].

### Gene expression in dinoflagellate mitochondria

Within the *K. micrum *EST survey, long cDNAs that encoded several mitochondrial genes or gene fragments (the longest being 5854 bp) were noted. By contrast, most mitochondrial cDNAs we recovered encoded a single gene, suggesting the longer transcripts may be rapidly processed into shorter molecules. Polycistronic transcripts up to 5.9 kb are also known from apicomplexan mtDNA, these are rapidly processed to short, single-gene transcripts [38]. Interestingly, the polycistronic transcripts from *K. micrum *are not edited, indicating that RNA editing acts on the individual gene transcripts.

The use of alternative initiation codons in dinoflagellate mitochondrial genes is consistent with what is seen in the mitochondria of other alveolates. In *Plasmodium *species, *cox1 *and *cox3 *lack an in-frame ATG, and while *cob *does contain a ATG near the initiation site, it is uncertain whether initiation occurs at this site or upstream of it [24] (as in the case of dinoflagellate *cob*). ATT and ATA have been proposed as alternative initiator codons in *Plasmodium *species [39] (as well as some animal, fungal and algal mitochondrial genes [9,40]). Several mitochondrial genes from the ciliate *Tetrahymena pyriformis *also apparently use alternative initiation codons of the form ATN or NTG: in the case of *cob *an ATG within eight codons of the predicted N-terminus is apparently ignored, with GTG used in its place [41]. Thus, there are precedents for reliance on codons other than ATG for translation initiation within alveolates. Potential initiator ATN/NTG codons exist in all three *Karlodinium *mitochondrial genes; however, a broader survey of dinoflagellates or analysis of protein sequences will be necessary to identify the most likely candidates.

An absence of stop codons is more unusual. In *T. pyriformis *all mitochondrial protein-coding genes terminate with TAA [12]. TGA encodes tryptophan (as in several mitochondrial systems [9,42]) and TAG is simply not used. All three *Plasmodium *mitochondrial protein-coding genes also use TAA [24]. By contrast many dinoflagellate mitochondrial gene transcripts appear to lack any termination codon. With only a single known exception (a *cox3 *fragment from *Lingulodinium polyedrum *[16]), transcripts are oligoadenylated upstream of any of the standard termination codons, and RNA editing does not generate an in-frame stop. Further, in none of the transcripts is a sense codon uniquely localized in the 3' region in such a way as to suggest that it serves as an alternative terminator (as in [8,43]). The oligoadenylation of *K. micrum cox3 *mRNA does produces a UAA codon, as is also the case for *cox3 *transcripts for *A. carterae *and *O. marina *[21], that suggests that *cox3*, unlike *cox1 *or *cob*, might utilize conventional stop. Such a mechanism for reconstituting a functional UAA is known to occur in some mammalian mitochondrial transcripts [40].

It is unclear how the mitochondrial translation machinery might cope with the absence of termination codons. Release factors that are essential for disassembly of the ribosome usually recognize specific codons, so the absence of these codons could block ribosome disassembly. There are precedents in other mitochondrial systems for the lack of termination codons: transcripts of two plant mitochondrial genes have been shown to be oligoadenylated upstream of in-frame stops [44]. Proteins encoded by both of these genes can be detected, indicating that the corresponding transcripts are successfully translated. In human mitochondria, a rare mutation has been shown to ablate a stop codon, and yet the corresponding protein is still detectable is these cell lines [45]. Eubacteria are known to be able to rescue damaged mRNA molecules that have lost their termination codon by use of a specialized RNA with properties of both a tRNA and an mRNA [46]. These so-called tmRNAs restart protein synthesis by providing a terminal mRNA section that encodes a functional stop codon. It has been speculated that an equivalent system might be used in plant and animal mitochondrial systems where mRNAs lack stop codons [44,45]. Indeed, tmRNA-like RNA species have been identified in the mitochondria of jakobid flagellates such as *R. americana*; however, these RNAs lack the terminal mRNA-like segment of a conventional tmRNA [47]. Moreover, the C-terminal tag provided by a tmRNA normally targets the modified protein for degradation rather than for function [48]. Whatever the actual mechanism of translation termination in dinoflagellate mitochondria, it appears to present a clear difference with respect to protein synthesis termination in ciliate and apicomplexan mitochondria.

Lastly, we have found a likely case of trans-splicing of dinoflagellate mitochondrial transcripts, which adds a further layer of complexity to genome organization and expression in these organelles. While we cannot conclusively eliminate the possibility of a complete *cox3 *coding sequence in dinoflagellates we have not been able to detect an intact gene. This negative result is consistent with all other studies to date, which report only partial *cox3 *sequences from five different dinoflagellate taxa [16-18,20,28] (note that the *A. catenella cox3 *is reported as complete [17], but it lacks approximately 300 nucleotides compared with homologs in other dinoflagellates and in apicomplexans). All available data from genomic fragments and transcripts suggest that the complete *cox3 *transcript is generated by trans-splicing. Such trans-splicing has not been reported for either apicomplexan or ciliate mitochondria. In ciliates *nad1 *is split into two segments [12] but they are independently transcribed, and there is no evidence of splicing of the corresponding transcripts to create a continuous, complete *nad1 *ORF [41]. Trans-splicing occurs in plant mitochondria [49,50], but in these cases the coding breakpoints are flanked by group II intron elements, which form secondary structures that mediate the splicing events. We have no evidence of group II introns in dinoflagellate mtDNA, but we do note that the intergenic sequences contain numerous inverted repeats consistent with extensive secondary structure, which might conceivably facilitate splicing events. The unique nature of the dinoflagellate trans-splicing is also evident from the inclusion of five A residues at the splice boundary that appear to derive from the oligo(A) tail of the upstream fragment. The removal of any downstream sequence by oligoadenylation prior to splicing argues against the involvement of a cis-acting element such as a group II intron in the splicing process. It is conceivable that oligoadenylation of the short 5' *cox3 *transcript could serve as a degradation signal for these short transcripts, as has been observed in human mitochondria [51]. However, lack of a complete *cox3 *coding sequence, coupled with the fact that the site of oligoadenylation corresponds with the break in coding sequence of 5' and 3' *cox3 *portions, suggests that the short *cox3 *transcripts are important intermediates in the generation of the complete *cox3 *transcripts.

### RNA editing

The RNA editing observed in *K. micrum cox1*, *cob *and *cox3 *mRNAs is consistent with the level and type of editing observed in *cox1 *and *cob *mRNAs in other dinoflagellate species [18-20], with the exception that *cox3 *is even more heavily edited than either of *cox1 *or *cob*. While some editing sites are conserved, others are unique to certain taxa, suggesting that new editing sites are constantly evolving in dinoflagellates. In this study we also found evidence in *K. micrum *of editing of rRNA fragments LSUG and LSUA. RNA editing of *A. catenella *LSUE has also recently been reported [17]. At present the data are insufficient to assess the conservation of rRNA editing sites among taxa; however, two inferred editing sites in *A. catenella *LSUE are not edited in *K. micrum*, suggesting that rRNA editing sites are constantly evolving as with those in protein-coding genes.

Whether RNA editing plays some functional role in dinoflagellate mitochondria is unclear. From analysis of protein-coding genes in several dinoflagellates, Lin et al [19] noted that the majority of editing events are to either a C or G, thus generating a net reduction in A+U content from the bias of ~70% for the coding sequences. We observe this trend also in *Karlodinium *protein-coding sequences. This re-tailoring of mRNA sequences might better accommodate the suite of nucleus-encoded tRNAs that are likely imported from the cytoplasm, and which typically participate in the decoding of nucleus-encoded mRNAs having a more balanced A+U content [19]. Ribosomal RNA is also sensitive to A+U content, with secondary structure elements such as hairpin loops better stabilized by G-C than by A-U pairs; thus, helical regions tend to be relatively more G+C rich than other rRNA domains. While the available data for rRNA editing are limited (14 editing sites), it is interesting that the editing types in rRNAs have an overall neutral impact on A+U content. Indeed the A+T content of mitochondrial genomic sequence specifying rRNAs is already much reduced (56%) compared to that of the protein-coding genes. This observation might add weight to the notion that editing helps correct (at the RNA level) the A+T skew of protein-coding genes.

The mechanism of RNA editing in dinoflagellate mitochondria is also unknown; however the possibility of a guide RNA (gRNA)-assisted mechanism, similar to that employed in trypanosomatid mitochondria [52], has recently been suggested [20]. Nash et al [20] report that gene fragments encoded in mitochondria sometimes encode the 'corrected' nucleotide at an inferred editing site (in 6 out of 25 sites for which they had data). Thus such fragments could encode templates that direct the editing events of full-length transcripts. We analyzed the *K. micrum *data for similar evidence of post-edited nucleotides represented in gene fragments. From five fragments (representing unambiguously truncated genes) that span 71 editing sites across the three protein-encoding genes, only one site in one of the fragments corresponds to a 'corrected' nucleotide seen in cDNAs at an inferred editing site (nucleotide 30 in the *cob *gene). An independent copy of the *cob *genomic sequence verified that this nucleotide difference is genuine (not a PCR error). Hence this might represent an example of an editing template in *K. micrum*; however, if gRNAs are responsible for all editing events, a very large number of additional fragments must exist to direct the remainder of the changes. Clearly further work is required to shed light on the mechanism of RNA editing in dinoflagellates.

### Future directions

A key question that remains is whether the observed diversity of dinoflagellate mitochondrial genes, gene fragments, and repetitive elements derives from a single mtDNA molecule or from multiple chromosomes. A similar scenario of mitochondrial genes occurring as multiple copies and fragments is seen in the ichthyosporean *A. parasiticum*, a unicellular organism closely related to animals [4]. In this protist, several hundred small linear chromosomes constitute the mitochondrial genome, each encoding a smattering of genes and partial genes. Diplonemids, members of the phylum Euglenozoa, also contain fragmented genes on separate circular mitochondrial chromosomes [6]. It is unknown whether either of these unusual situations applies to the organization of the dinoflagellate mitochondrial genome; however, in this regard we make two preliminary observations. One is that long-range PCR was unable to generate longer contiguous sequences linking the many mtDNA elements we report in this study. Rather, additional short unique gene linkages were obtained, and it is clear that we have yet to sample the full diversity of gene combinations. Secondly, the presence of individual genes in partial tandem repeats (see Figure 3B, vi and viii) is consistent with minicircles, as seen in dinoflagellate plastid genomes [53]. If these cases represent true minicircles, we have been unable to amplify a corresponding sequence to close these circles (note that Figure 3B, vi and vii contain unique sequence relative to vi and viii, respectively). It is also possible, of course, that the tandem repeats that we observe are simply a consequence of further recombination events, and the high diversity of gene combinations.

## Conclusion

A greater depth of sampling of dinoflagellate mitochondrial DNA and mRNA has provided a clearer view of a complex genome and many peculiarities of gene expression. We find that the dinoflagellate mitochondrial genome shares several features in common with the mtDNA of its apicomplexan sister lineage, but also many novel characteristics. Features in common for the two lineages are: (1) a very high level of gene relocation from the mitochondrion, (2) extensive rRNA gene fragmentation and dispersal, and (3) use of non-standard initiation codons. Features unique to dinoflagellates are: (1) gene copy number expansion and reorganization, (2) loss of stop codons from protein-coding genes, (3) mRNA trans-splicing, and (4) RNA editing of protein-coding and rRNA transcripts. These data demonstrate a remarkable burst of organelle genome evolution in dinoflagellates following divergence from Apicomplexa, and also challenge our understanding of the mechanistic details of genome maintenance and expression, most notably translation termination.

## Methods

### Cell culture, nucleic acid extraction, and mtDNA cloning

*C. cohnii *cells were cultured and nucleic acids extracted as previously described [54].*K. micrum *and *H. triquetra *were cultured as previously described [22,26] and genomic DNA was extracted using the DNEasy Plant Minikit (Qiagen, Hilden, Germany). For *C. cohnii*, a fraction was enriched in mtDNA by isolating mitochondria via subcellular fractionation. This fraction was hydrolyzed with *Eco*RI and ligated into pBluescript KS+ (Stratagene, Cedar Creek, Texas, USA), following which plasmids were transformed into competent *E. coli *cells [55]. Hybridization probes 'cob' and 'cox3' (see Southern blot analysis, below) were used to identify positive clones by hybridization of colony lifts [56]. For *K. micrum *PCR was used to amplify mtDNA fragments using oligonucleotides (20–22 nucleotides) designed from mitochondrial genes identified from an EST survey [22] using TBestDB [57]. PCR products were cloned into pGEM^® ^-T Easy vector (Promega, Madison, Wisconsin, USA) and fully sequenced. Additional primers were designed from sequence derived from these products. Analysis of DNA sequences was performed with the software package Sequencher™ 4.2.2 (Gene Codes Corporation, Ann Arbor, Michigan, USA). Protein alignments were made with the software packages Clustal X [58] and McClade (Sinauer Associates, Massachusetts, USA). New sequences have been submitted to GenBank (GenBank accession numbers EF442995–EF443047, and AM773790–AM773803).

### Southern blot analysis

Five hybridization probes were generated using PCR and restriction products as template. The 'cob' probe (753 nucleotides), corresponding to positions 1386–2138 in pcb#2 and encompassing most of the *cob *reading frame, was amplified by PCR using cob51 (5'-CTGTGGTCCAGATATCTTTC-3') and cob296 (5'-CTTCTAATGAATTATCTG-3') primers. 'cb1' (430 nucleotides) was generated by PCR from pcb#7 using primer sets P51 (5'-CTATCTAAATCCTATAAACAATG-3'; positions 2411–2433) and P25 (5'-AAGGATTTGGTTTCTTGATG-3'; positions 2821–2840) and 'cb3' (716 nucleotides) from pcb#2 using primer P50 (5'-CTGCCAGAGAATTATTGGTTAAC-3') and M13 reverse vector-based primer. 'cox3' was generated by *Bam*HI hydrolysis of a *cox3*-containing clone previously prepared. The deduced amino acid sequence of this 300-nt fragment exhibited a high degree of identity with that of cytochrome oxidase subunit 3 (Cox3) in *P. falciparum *(amino acids 272–289). All of these fragments were purified from gels and used as templates in random hexamer radiolabelling as previously described [54]. A final Southern hybridization probe, 'rnl' (specific for LSUG), consisted of an 18-mer oligonucleotide (5'-GGTTAGAAACTGTCGCTG-3') that was 5' ^32^P-end-labelled [56]. Unincorporated isotope was removed by spin chromatography using a Sephadex G-25 MicroSpin™ column (Pharmacia,,,,,,, London, UK). Southern hybridization and filter washing conditions were as previously outlined [54] using RNase A-treated DNA samples to eliminate any RNA contamination.

### Transcriptional analysis

*K. micrum *and *H. triquetra *transcripts were inferred from cDNAs prepared as previously described for EST surveys [22,26]. Complete sequences were generated from cDNAs maintained as frozen *E. coli *clones. RT-PCR was used to amplify mRNA sequences not represented in the initial EST survey (e.g. full length *cox1*). The 3' ends of transcripts were inferred from oligoadenylation sites.

For *C. cohnii*, 3'-end mapping of rRNAs was performed using 3'-RACE. Briefly, isolated mtRNA was incubated with recombinant yeast poly(A) polymerase (USB) and 0.5 mM CTP for 20 min followed by a 10 min incubation with 0.5 mM ATP using the same conditions as previously outlined [59,60]. cDNA synthesis was performed using AMV reverse transcriptase (Promega) with an oligo(dT) primer (5'-AATAAAGCGGCCGCGGATCCAATTTTTTTTTTTTTTTTVN-3') [61] following manufacturer's protocols. The cDNA was used in PCR amplification with primers P4 (5'-AATAAAGCGGCCGCGGATCCAA-3') and either LSUG4 (5'-AGAAGATTCCATTGGAAG-3') for LSUG, or LSUE4 (5'-AAGGTAGNNNAATTCCTTGATAGG-3') for LSUE. PCR amplification products were cloned into pT7Blue T-vector (Novagen) and sequenced. LSUE 5'-end sequence was generated by cDNA sequencing using primer LSUE2 (5'-TTCATGCAGGACGGARMTTACCC-3'. Ribosomal RNA sequences were manually fitted to the *Escherichia coli *secondary structure models [62] and the structure diagrams were drawn using the program XRNA (B Weiser and H Noller, personal communication).

## Abbreviations

nt, nucleotides; bp, basepairs; cDNA, complementary DNA; rRNA, ribosomal RNA; kb, kilobase; mtDNA, mitochondrial DNA; PCR, polymerase chain reaction; RT-PCR, reverse transcriptase polymerase chain reaction; RACE, rapid amplification of cDNA ends; LSU, large subunit; SSU, small subunit; ORF, open reading frame; EST, expressed sequence tag; tmRNA, transfer-messenger RNA; UTR, untranslated region.

## Competing interests

The author(s) declares that there are no competing interests.

## Authors' contributions

CJJ generated *K. micrum *and *H. triquetra *data, and drafted the manuscript. JEN generated *C. cohnii *data. MNS modeled rRNA secondary structures. PJK provided access to *K. micrum *and *H. triquetra *EST data and cDNA libraries and contributed to study conception. MWG contributed to study conception. RFW contributed to study conception and drafted the manuscript. All authors contributed to data analysis, manuscript revision and approved the final manuscript.
